# The A. D. S. E. Report

**Published:** 1888-01

**Authors:** 


### THE A. D. S. E. REPORT.

In "Current News" for this number will be found a letter from Prof. Peirce, of the Pennsylvania College of Dentistry, denying the correctness of certain statements in the report of the American Dental Society of Europe, as published in the last number of this Journal.

As soon as it could be put in type, proofs of that part of the report were forwarded to Dr. Miller. Had he been within reach, they would have been sent him before the publication. Immediately upon receiving them, he wrote us that the reporter had misconstrued his language somewhat, and said—

"The remarks I made were about as follows: Certain of the dental schools in America have succeeded in bringing the title D. D. S. into such bad repute that it is not only no honor to possess that degree, but its possessor carries with it the suspicion that the possessor is either a quack or an ignoramus, who, being unable to accomplish anything here, has either bought a diploma in America, or, what is really worse for the standing of the profession, has obtained it by a mere farce of an examination, regardless of previous education. You all know the present standing of the American dentist in Germany, and the endless annoyances that esteemed Americans have been put to by the war against them. I need not recapitulate them. Suffice it to say, that 'D. D. S.' and 'American Dentist' are adornments of which we often have occasion to feel ashamed, and which we cannot attach to our names without being eyed with suspicion. (Dr. Patton; *exactly!*) According to Petermann's Almanack, there were practicing in Germany, in 1885 (excluding Americans), graduates of the Pennsylvania Dental College, forty; the Philadelphia Dental College, thirty-four; the Baltimore Dental College, seventeen; the New York Dental College, fourteen. No other college had furnished more than three.

"From this list each one draws his own conclusions, though I should say that it might not be just to infer that the four colleges named are all necessarily guilty, or that they have lowered the standing of the dental profession in exactly the proportion indicated by the above figures. Among other things, the age of the college should be taken into consideration. Perhaps some of our younger colleges have not yet had time to show how fast they can turn out doctors."

It will be seen that Dr. Miller disclaims having said that they had a list of diplomas improperly granted, but that it was one of diplomas granted to practitioners in Germany other than Americans. The reporter understood him to give a list of foreign false diplomas, and so stated it. It was impossible for us to send proof of their remarks to speakers for correction, and we were forced either to suppress the report entirely or to publish it substantially as received.

Petermann's list of foreign graduates of American colleges was published in 1885, and must have been compiled from the returns of 1884. It was in that year that the INDEPENDENT PRACTITIONER published a series of articles upon "Dental Education," which made a decided commotion, and the immediate result was the formation of the Association of Dental College Faculties, and a long step toward a higher standard and a degree of uniformity in conferring diplomas. From that time we believe that the irregular granting of degrees was materially checked, if not altogether stopped. We have publicly offered to publish the particulars of any well-attested case of irregular graduation, if any such occurred, but none has been regularly brought to our notice, and our natural inference is that a practical reform has been brought about, and that henceforth American dentists abroad will have no cause for complaint. If such a case should come to their knowledge, their duty is to see that it be published to the world. It will take a long time to erase from the minds of foreign people the impression left by the disgraceful Delavan fraud, and the too lax rules of some reputable colleges in granting degrees to foreigners, but if the schools will, in the future, rigidly hold each other to the agreement of the Association of Dental College Faculties, the D. D. S. will be lifted from its present low condition in Europe.

We think that the extract from Dr. Miller's letter is a sufficient explanation and answer to the interrogatories of Prof. Peirce.

